# Supplementary material for: The interactome of intact mitochondria by cross-linking mass spectrometry provides evidence for coexisting respiratory supercomplexes
Source: Mol Cell Proteomics. 2017 Dec 8;17(2):216–32. doi: 10.1074/mcp.RA117.000470 (PMC5795388; doi:10.1074/mcp.RA117.000470)
Supplement: Supplemental Data [file supp_RA117.000470_133922_0_supp_23978_fzffwf.zip › spectra_annotation/mito_spectra_annotation/152-1-40-1-12-1.pdf]

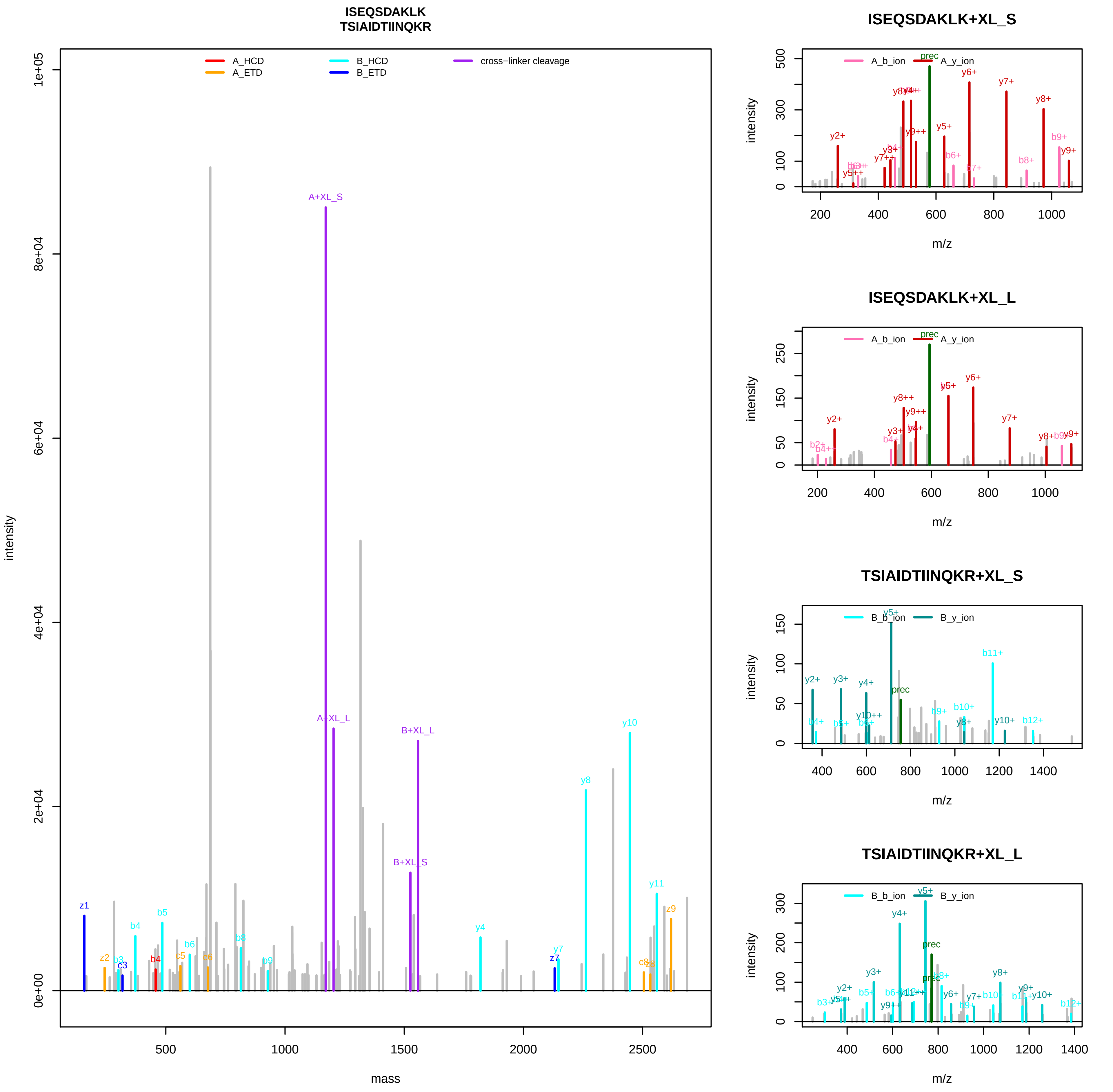
ISEQSDAKLK
TSIAIDTIINQKR
A_HCD
A_ETD
B_HCD
B_ETD
cross−linker cleavage
intensity
mass
0e+00
2e+04
4e+04
6e+04
8e+04
1e+05
500
1000
1500
2000
2500
A+XL_S
A+XL_L
B+XL_L
B+XL_S
z1
z2
b3
c3
b4
b5
b6
b8
b9
c5
c6
z7
y4
y7
y8
y10
y11
z9
ISEQSDAKLK+XL_S
ISEQSDAKLK+XL_L
TSIAIDTIINQKR+XL_S
TSIAIDTIINQKR+XL_L
A_b_ion
A_y_ion
B_b_ion
B_y_ion
prec
m/z
y2+
y5+
y6+
y7+
y8+
y9+
b8+
b9+
b6+
b7+
b11+
b12+
y10+
b10+
y4+
y3+
